# Attitude and influencing factors of patients with schizophrenia toward long-acting injections: A community-based cross-sectional investigation in China

**DOI:** 10.3389/fpubh.2022.951544

**Published:** 2022-10-10

**Authors:** Yiying Sun, Jie Tong, Ying Feng, Haiping Fang, Tao Jiang, Liping Zhao, Qiang Wang, Yi Yang

**Affiliations:** ^1^Clinical Research Center for Mental Disorders, Shanghai Pudong New Area Mental Health Center, School of Medicine, Tongji University, Shanghai, China; ^2^Mental Health Subcentre of Shanghai Pudong New Area Center for Disease Control and Prevention, Shanghai, China; ^3^Shanghai Yangjing Community Health Service Center, Shanghai, China; ^4^Shanghai Sanlin Community Health Service Center, Shanghai, China

**Keywords:** long-acting injections, LAIs, schizophrenia, community, China

## Abstract

**Background:**

Low prescription rates of antipsychotic long-acting injections (LAIs) may be a major challenge in the prevention and treatment of schizophrenia. However, there are few studies on the usage and attitude toward LAIs among community-based patients with schizophrenia.

**Methods:**

A large community-based cross-sectional investigation was conducted among 6,336 patients with schizophrenia from Shanghai, China from March 1 to June 30, 2021. The structured Attitude and Status toward Treatment of Community Patients with Schizophrenia Questionnaire (AST-CSQ) was used to investigate the attitude and influencing factors of community-dwelling patients with schizophrenia toward LAIs.

**Results:**

Among the 6,336 participants, the average age was 49.28 ± 11.23. The rate of agreement to LAI antipsychotics among participants was 3.16% (*n* = 200). The family financial resources, care ability, and disease course of the LAIs group were less than those of the non-LAIs group. However, the LAIs group had higher immediate family guardianship, social activity, previous hospitalization, number of hospitalization, outpatient adherence, previous antipsychotic use, antipsychotic adherence, and attitude toward oral antipsychotics than the non-LAIs group, with significant differences between the two groups (*p* < 0.05). Furthermore, age (β = −0.036, OR 0.964, 95% CI 0.947–0.982), marital status (β = 0.237, OR 1.267, 95% CI 1.002–1.602), care ability (β = 0.709, OR 2.032, 95% CI 1.437–2.875), outpatient adherence (β = −0.674, OR 0.510, 95% CI 0.358–0.725), antipsychotic adherence (β = 0.920, OR 2.509, 95% CI 1.092–5.764), and attitude toward oral antipsychotics (β = −1.357, OR 0.258, 95% CI 0.103–0.646) were significant predictors of attitude toward LAI antipsychotics (*p* < 0.05).

**Conclusions:**

The community-dwelling patients with schizophrenia in China had a low willingness to use LAIs. Patients of a younger age, more hospitalizations, and a shorter course of disease were prone to be more willing to accept LAIs. The patients' age, marital status, care ability, outpatient adherence, antipsychotic adherence, and attitude toward oral antipsychotics were important predictor of patients' attitudes toward LAIs. Under the global deinstitutionalized management model of mental disorders, these results highlight an urgent problems for public mental health service providers and policy-makers and provide more solutions for them.

## Background

The prevention of relapse and hospitalization in individuals with schizophrenia is a major public mental health challenge (1). To date, the problems of multiple relapses in the course of schizophrenia are difficult to solve and have attracted extensive attention worldwide (2, 3). According to a WHO report in 2018, the number of patients with schizophrenia in the world reached 24 million, with a prevalence of 3.8 to 8.4 ‰ and a 10-year recurrence rate of 75% (4). In Europe, estimates of the excess costs of relapse in schizophrenia range from $8665 to 18,676 over periods of 6–12 months, while in the US, it can reach $16,000 to 33,000 over a period of 6–15 months (5). Most studies have shown that poor treatment adherence has become an important factor in multiple relapses of schizophrenia (6, 7).

Long-acting injectable (LAI) antipsychotics have been shown to improve treatment adherence in patients with schizophrenia and decrease the rate of relapse and hospitalization, which makes them superior to their oral counterparts in this regard (8). LAIs are a new medical controlled-release technology that can reduce the number of times patients take medicine, so they can effectively improve medication adherence (9). Subotnik et al. (10) showed that the relapse rate and/or psychotic exacerbation of first episode of schizophrenia was lower for the LAIs group than for the oral group, and the relative risk was reduced by 84.7%. A meta-analysis of 147 studies suggests that LAIs are more beneficial than oral antipsychotics in terms of insight, efficacy, effectiveness, safety and quality of life (11).

However, the clinical use rate of LAIs has not reached expectations, especially for community-dwelling patients with schizophrenia, which puts forward new problems for public mental health (12). In European countries, the second-generation antipsychotic LAI prescription rate is lower than 30%, while that in the United States is only 10% (13). The limited use of LAIs appears to be related to the negative attitudes of clinicians and inpatients toward this treatment (14). A total of 17.6% of physicians declared feeling more pressure to offer LAI antipsychotics than oral antipsychotics (15). Most patients believed that LAIs were less effective, more expensive and had more serious side effects than oral antipsychotics (16).

At present, there are few studies on the usage of and attitude toward LAIs among community-dwelling patients with schizophrenia. We do not know the factors related to the low use of LAIs in community-dwelling patients. According to the data of the mental health center of the Chinese Center for Disease Control and Prevention, the number of patients with schizophrenia in China has exceeded 6.4 million (17). In the future, the rehabilitation of schizophrenia will be gradually deinstitutionalized and the community rehabilitation model will be realized (18). This large community-based cross-sectional investigation explored the attitude and influencing factors of Chinese community-dwelling patients with schizophrenia toward LAIs.

## Methods

### Study design

This study was conceptualized as a community-based cross-sectional investigation. It was conducted by the Mental Health subcentre of Shanghai Pudong New Area Center for Disease Control and Prevention, Shanghai Pudong New Area Mental Health Center, Tongji University School of Medicine, which has been dedicated to fully functioning community mental health prevention and research since 2010. The sample size of the study was calculated using the PASS version 21.0.3 (NCSS LLC, Utah, USA), a sample size and power analysis software. Taking the significance level (α) was 0.05, the confidence level (1-α) was 0.95, the allowable error (δ) was 0.03, and the proportion (p) was 0.5, and the calculated sample size was 1,098. Considering the special population, the loss rate was set at 40%, the calculated sample size was at least 1,830. A total of 10,305 paper informed consent forms and questionnaires were distributed to patients with schizophrenia in 23 residential districts and 32 community health service centers in Pudong New Area, Shanghai. A total of 6,336 individuals agreed to participate in and complete the questionnaires from March 1 to June 30, 2021. The attrition rate of participants was 38.52%. This study was ethically reviewed by the Research Ethics Committee of the Shanghai Pudong New Area Mental Health Center and Tongji University Mental Health Center.

### Participants

All participants were community-based psychiatric patients from Pudong New Area, Shanghai from March 1 to June 30, 2021. The following inclusion criteria were employed: (1) patients meeting the DSM-5 (19) diagnostic criteria for schizophrenia; (2) age ≤ 65 years old; (3) certain visual and auditory resolution without cognitive disorders; (4) currently living in the community and not hospitalized; and (5) both the participants and guardians agreed to participate in the investigation and signed the informed consent. The following exclusion criteria were employed: (1) severe visual or hearing impairment, physical disability, extracranial trauma or history of surgery; (2) obvious excitement impulse, serious negativity, self-harm or suicidal ideation; (3) dementia or developmental delay diagnosed as behavioral disorder; and (4) participants or guardians who did not sign the informed consent form, or withdrew halfway.

### Outcomes

#### Attitude and status toward treatment of community patients with schizophrenia questionnaire

This investigation was conducted using the self-made AST-CSQ. To ensure the quality and rationality of the survey, three measures were taken to finalize the questionnaire. First, referring to the Medication Adherence Rating Scale (MARS) (20, 21), Drug Attitude Inventory (DAI) (22), and Multidimensional Scale of Perceived Social Support (MSPSS) (23), the expert consultation determined the questionnaire items. Second, after three rounds of expert committee argumentation, the first version of the questionnaire is determined. Third, the revised version of the questionnaire were determined according to the pre- survey problems. The expert committee consisted of four psychiatrists, four public health experts, two psychopharmacologists, one health economists, and one health statistician.

The structured AST-CSQ was used to investigate the attitude and status toward treatment of community patients with schizophrenia. The questionnaire consists of four sections and twenty questions (Table 1). The first part is demographic characteristics, including age, gender, marriage, residence, education, and occupation. The second part deals with the social support of the respondents, including economic status, medical expenses, guardians, care ability, and social activities. The third part is related to the treatment experience, including course of disease, number of episodes, inpatient or outpatient experience. The last part focuses on attitude toward oral and long-acting injectable antipsychotics (24). In addition, attitudes toward antipsychotics refer to agreement or disagreement to receive oral or long-acting injectable antipsychotics. The questionnaire was completed by 32 community psychiatric public health physicians through face-to-face interviews with respondents. All investigators were trained for consistency.

**Table 1:** Attitude and status toward treatment of community patients with schizophrenia questionnaire (AST-CSQ).

**Section 1 (Demographic characteristics)**
1. How old are you?
2. What is your gender?
3. What is your marital status?
4. Are you a registered residence in Shanghai?
5. What is your education?
6. What is your occupation?
**Section 2 (Social support)**
7. How is your family's financial resources?
8. Where does your medical expenses come from?
9. What is your relationship with guardians?
10. How about the guardian's care ability?
11. Do you often involve in social activities or housework?
**Section 3 (Treatment experience)**
12. How many years is your course of disease?
13. How many times are the accidents caused by illness?
14. Have you ever been hospitalized?
15. How many times have you been hospitalized?
16. Do you have regular outpatient visits?
**Section 4 (Attitude toward antipsychotics)**
17. Have you ever taken antipsychotics?
18. Could you take your medicine on time according to the doctor's advice every day?
19. Would you like to take oral antipsychotics?
20. Would you like to take long-acting injectable antipsychotics?

### Data analysis

Data were analyzed using SPSS version 25.0 statistical software (SPSS, Inc., Chicago, IL, USA). We first identified the patients' attitude acceptance rate of LAIs and divided them into an LAIs group and a non-LAIs group. Descriptive analysis was performed for sociodemographic data. All continuous variables are first tested for normality, and the variables with normal distribution were described by the mean mean ± standard deviation. The continuous variables were compared between groups using the independent samples *t* test. For classified data, the frequency (percentage) was used for statistical description, and the chi-square (χ^2^) test was used for intergroup comparison. Binary logistic regression analysis was used to explore the predictors of influencing factors for attitude toward LAI antipsychotics. The difference was statistically significant at *p* < 0.05.

## Results

### Demographic characteristics

The demographic characteristics of community-based patients with schizophrenia, including age, gender, marital status, residence, education and occupation, are shown in Section Demographic characteristics of Table 2. Among the 6,336 participants, the average age was 49.28±11.23 years, and the proportion of females was higher (52.53%). In addition, most of the respondents were unmarried (50.45%), registered a residence in the city (96.65%), had a junior high school education (43.58%), and were unemployment (52.10%). Respondents of the LAIs group were younger and less employed than those of the non-LAIs group, and there were significant difference between the two groups (*p* < 0.001). But there was no significant difference in other control variables of demographic characteristics (*p* > 0.05).

**Table 2:** Demographic characteristics, social support, treatment experience, and attitude toward antipsychotics of participants.

**Variable**	**Overall (*n* = 6,336)**	**LAIs group (*n* = 200)**	**non-LAIs group (*n* = 6,136)**	**t _/_ x_2_**	***p***
**Section 1 (Demographic characteristics)**				
Age in years (mean ± SD)	49.28 ± 11.23	44.65 ± 11.09	49.43 ± 11.20	5.945	<0.001**
Gender, *n* (%)				0.340	0.560
Male	3,008 (47.47%)	99 (49.50%)	2,909 (47.41%)		
Female	3,328 (52.53%)	101 (50.50%)	3,227 (52.59%)		
Marital status, *n* (%)				3.258	0.354
Unmarried	3,197 (50.45%)	106 (53.00%)	3,091 (50.40%)		
Married	2,619 (41.34%)	74 (37.00%)	2,545 (41.50%)		
Divorce	456 (7.20%)	19 (9.50%)	437 (7.10%)		
Widowed	64 (1.01%)	1 (0.50%)	63 (1.00%)		
Residence, *n* (%)				3.181	0.204
Registered residence	6,124 (96.65%)	189 (94.50%)	5,935 (96.72%)		
Non-registered residence	212 (3.35%)	11 (5.50%)	201(3.28%)		
Education, *n* (%)				11.519	0.074
Primary school and below	442 (6.98%)	13 (6.50%)	429 (6.99%)		
Junior high school	2,761 (43.58%)	93 (46.50%)	2,668 (43.48%)		
Senior high school	2,049 (32.34%)	49 (24.50%)	2,000 (32.59%)		
Junior college or above	1,084 (17.10%)	45 (22.50%)	1,039 (16.94%)		
Occupation, *n* (%)				21.789	<0.001**
Student	80 (1.26%)	4 (2.00%)	76 (1.24%)		
Employed	1,597 (25.21%)	49 (24.50%)	1,548 (25.23%)		
Retire	1,358 (21.43%)	25 (12.50%)	1,333 (21.72%)		
Unemployed	3,301 (52.10%)	122 (61.00%)	3,179 (51.81%)		
**Section 2 (Social support)**
Family financial resources, *n* (%)				4.932	0.026*
Poor	976 (15.40%)	42 (21.00%)	934 (15.22%)		
Good	5,360 (84.60%)	158 (79.00%)	5,202 (84.78%)		
Source of medical expenses, *n* (%)				3.913	0.141
Own expense	928 (14.65%)	36 (18.00%)	892 (14.54%)		
Medical insurance	5,222 (82.42%)	155 (77.50%)	5,067 (82.58%)		
Others	186 (2.93%)	9 (4.50%)	177 (2.88%)		
Guardian relationship, *n* (%)				8.089	0.044*
Parents or children	3,455 (54.53%)	126 (63.00%)	3,329 (54.25%)		
Spouse	1,839 (29.02%)	51 (25.50%)	1,788 (29.14%)		
Others	1,042 (16.45%)	23 (11.50%)	1,019 (16.61%)		
Care ability, *n* (%)				22.778	<0.001**
Poor	730 (11.52%)	44 (22.00%)	686 (11.18%)		
Good	5,585 (88.14%)	156 (78.00%)	5,429 (88.48%)		
None	21 (0.34%)	0 (0.00%)	21 (0.34%)		
Social activities, *n* (%)				15.681	<0.001**
Involve in community activities	891 (14.06%)	29 (14.50%)	862 (14.05%)		
Only involve in housework	4,515 (71.26%)	161 (80.50%)	4,354 (70.96%)		
Not involve in any activities	930 (85.32%)	10 (5.00%)	920 (14.99%)		
**Section 3 (Treatment experience)**
Course of disease (mean ± SD)	18.05 ± 12.11	14.30 ± 10.06	18.17 ± 12.15	5.320	<0.001**
Number of accidents (mean ± SD)	0.03 ± 0.46	0.06 ± 0.40	0.03 ± 0.46	0.675	0.500
Hospitalization, *n* (%)				42.218	<0.001**
Yes	815 (12.86%)	56 (28.00%)	759 (12.37%)		
No	5,521 (87.14%)	144 (72.00%)	5,377 (87.63%)		
Number of hospitalizations (mean ± SD)	0.67 ± 1.51	1.15 ± 1.91	0.66 ± 1.49	3.674	<0.001**
Outpatient adherence, n (%)				51.851	<0.001**
Regular	4,233 (66.80%)	177 (88.50%)	4,056 (66.10%)		
Irregular	492 (7.77%)	15 (7.50%)	477 (7.77%)		
Never	1,611 (25.43%)	8 (4.00%)	1,603 (26.13%)		
**Section 4 (Attitude toward antipsychotics)**
History of antipsychotic use				5.491	0.019*
Yes	6,172 (97.4 %)	200 (100.00%)	5,972 (97.32%)		
No	164 (2.6%)	0 (0.00%)	164 (2.68%)		
Antipsychotics adherence, *n* (%)				27.434	<0.001**
Regular	5,173 (81.64%)	185 (92.50%)	4,988 (81.29%)		
Irregular	274 (4.32%)	12 (6.00%)	262 (4.27%)		
Never	917 (14.04%)	31 (15.50%)	886 (14.44%)		
Attitude toward oral antipsychotics, *n* (%)				29.858	<0.001**
Agree	5,104 (80.56%)	190 (95.00%)	4,914 (80.08%)		
Disagree	889 (14.03%)	3 (1.50%)	886 (14.44%)		
Uncertain	343 (5.41%)	7 (3.5%)	336 (5.48%)		
Attitude toward LAI antipsychotics, *n* (%)				14.37	<0.001**
Agree	200 (3.16 %)	200 (100.00%)	0 (0.00%)		
Disagree	6,136 (96.84%)	0 (0.00%)	6,136 (100.00%)		

LAIs, Long-Acting Injections. ^*^p < 0.05. ^**^p < 0.01.

### Attitude toward LAIs

The social support, treatment experience, and attitude toward antipsychotics among participants from the overall and the two groups in this study are reported in Section Social support to Attitude toward antipsychotics of Table 2. The rate of agreement to LAI antipsychotics among participants was 3.16% (*n* = 200), and there was a significant difference between the two groups (χ^2^ = 14.37, *p* < 0.001). The family financial resources, care ability, and disease course of the LAIs group were less than those of the non-LAIs group, and there were significant differences between the two groups (*p* < 0.05). However, the LAIs group had higher immediate family guardianship, social activity, previous hospitalization, number of hospitalization, outpatient adherence, previous antipsychotic use, antipsychotic adherence, and attitude toward oral antipsychotics than the non-LAIs group, with significant differences between the two groups (*p* < 0.05). There was no significant difference in the source of medical expenses and the number of accidents (*p* > 0.05).

### Regression analysis

The independent variables were different influencing factors of treatment attitude among community-based patients with schizophrenia; attitude toward LAI antipsychotics was the dependent variable for binary logistic regression analysis (Table 3). Age (β = −0.036, OR 0.964, 95% CI 0.947–0.982), marital status (β = 0.237, OR 1.267, 95% CI 1.002–1.602), care ability (β = 0.709, OR 2.032, 95% CI 1.437–2.875), outpatient adherence (β = −0.674, OR 0.510, 95% CI 0.358–0.725), antipsychotic adherence (β = 0.920, OR 2.509, 95% CI 1.092–5.764), and attitude toward oral antipsychotics (β = −1.357, OR 0.258, 95% CI 0.103–0.646) were significant predictors of attitude toward LAI antipsychotics (*p* < 0.05).

**Table 3:** Binary logistic regression analysis of different influencing factors on attitude toward LAIs.

**Variable**	**β**	**Std. Error**	**Wald**	***P***	**OR (95% CI)**
Age in years	−0.036	0.009	15.296	<0.001**	0.964 (0.947~0.982)
Gender	−0.132	0.153	0.745	0.388	0.876 (0.650~1.182)
Marital status	0.237	0.120	3.893	0.048*	1.267 (1.002~1.602)
Residence	−0.048	0.160	0.092	0.761	0.953 (0.697~1.302)
Education	−0.106	0.073	2.112	0.146	0.899 (0.779~1.038)
Occupation	−0.120	0.074	2.599	0.107	0.887 (0.767~1.026)
Family financial resources	−0.301	0.189	2.536	0.111	0.740 (0.510~1.072)
Source of medical expense	0.131	0.146	0.809	0.368	1.140 (0.857~1.516)
Guardian relationship	0.003	0.092	0.001	0.973	1.003 (0.837~1.202)
Care ability	0.709	0.177	16.050	<0.001**	2.032 (1.437~2.875)
Social activities	−0.077	0.184	0.176	0.675	0.926 (0.644~1.327)
Course of disease	−0.014	0.009	2.483	0.115	0.986 (0.970~1.003)
Number of accidents	0.024	0.133	0.032	0.857	1.024 (0.789~1.329)
Hospitalization	−0.229	0.190	1.451	0.228	0.795 (0.548~1.154)
Number of hospitalizations	0.085	0.045	3.496	0.062	1.088 (0.996~1.189)
Outpatient adherence	−0.674	0.180	14.059	<0.001**	0.510 (0.358~0.725)
Antipsychotics adherence	0.920	0.424	4.701	0.030*	2.509 (1.092~5.764)
Attitude toward oral antipsychotics	−1.357	0.470	8.348	0.004**	0.258 (0.103~0.646)

^*^p < 0.05. ^**^p < 0.01.

## Discussion

We examined the attitude and influencing factors of community-based patients with schizophrenia toward LAIs by the AST-CSQ, which is based on a large community-based cross-sectional investigation. To date, our study is the largest survey of community patients-dwelling with schizophrenia, and it can address the relevant factors of patients' attitudes toward LAIs more comprehensively, which is an original research direction. We found that Chinese community-based patients with schizophrenia had a low willingness to use LAIs, only 3.16%. There is a huge gap, with a 30% prescription rate of LAIs in the clinical setting of European countries and an 18.2% utilization rate for outpatients in Japan (13, 25). Regarding attitudes toward oral antipsychotics, 80.56% of the participants agreed with their use. This is also similar to the results of a study conducted by Grover et al. (26), 78.8% of patients in India are still willing to choose oral tablets.

On the other hand, this study also found that there were significant differences in age, hospitalization, and course of disease between the LAIs group and the non-LAIs group, that is, patients of a younger age, more hospitalizations, and a shorter course of disease were prone to be more willing to accept LAIs. This may be related to the high demand for social function recovery of patients or guardians of a younger age, more hospitalizations, or a short course of disease and their obvious desire to reduce recurrence (27). In contrary, older patients with schizophrenia suffer from long-term disease, and their social function and insight are greatly damaged, which seriously affects their expectation of the efficacy of LAIs. These results were consistent with the study of Xiao et al. (28). Perhaps this suggests that it is necessary to develop a personalized intervention plan for the population of this age and disease course structure and carry out targeted medication guidance (29).

Furthermore, the attitude toward and willingness to use LAIs of community-based patients with schizophrenia will also be affected by the characteristics of family monitoring and the coexistence of disease outcome (30, 31). Our study showed that patients' age, marital status, care ability, outpatient adherence, antipsychotic adherence, and attitude toward oral antipsychotics were important predictor of patients' attitudes toward LAI antipsychotics. Consistent with the results of this study, Grover et al. found that patients with low antipsychotic adherence were less willing to accept LAI treatment (26). Interestingly, most studies have found that patients with poor care ability were more likely to choose LAI treatment (32). These patients hope to reduce the cost of monitoring by reducing the number of oral antipsychotics used (33, 34). The antipsychotic adherence of patients with antipsychotics directly affects their motivation and understanding in choosing LAIs (35, 36).

Understanding the attitudes and influencing factors of real-world community-dwelling patients toward LAIs and formulating targeted drug publicity plans may be an effective method for mental health control and prevention of schizophrenia in the future (37). Most previous studies were based on inpatients or outpatients with clinical schizophrenia. In contrast, our study was on a natural sample population of community-based patients (38). Because patients' attitudes toward LAIs are easily influenced by psychiatrists, this may lead to study bias (39). Grover et al. (40) suggested that psychiatrists' attitudes toward LAIs play an important role in the prescription rate of LAIs. Communicating and explaining according to the characteristics of different patients can effectively change their attitudes toward LAIs (41). In the context of deinstitutionalization of global mental disorder management, mental health managers and psychiatrists should participate together to optimize mental health management techniques and strengthen health education for patients with mental disorders in the community (42). This could form a more open relationship and stronger mutual trust to improve patients' support for long-term drugs and reduce the risk of disease recurrence.

## Limitations

We also note several limitations. First, we were unable to conduct a nationwide multicentre study because of the COVID-19 pandemic. Second, we did not expand the use of measurement tools to measure psychiatric symptoms or social support systems. Future studies should explore the final outcome of community-based patients with schizophrenia using oral antipsychotics and LAIs by a long-term longitudinal study. This could provide more treatment options for clinical psychiatrists or public health practitioners to prevent the recurrence of mental diseases.

## Conclusion

In this study, community-dwelling patients with schizophrenia in China had a low willingness to use LAIs. Patients of a younger age, more hospitalizations, and a shorter course of disease were prone to be more willing to accept LAIs. The patients' age, marital status, care ability, outpatient adherence, antipsychotic adherence, and attitude toward oral antipsychotics were important predictor of patients' attitudes toward LAIs. Under the global deinstitutionalized management model of mental disorders, these results highlight an urgent problems for public mental health service providers and policy-makers and provide more solutions for them.

## Data availability statement

The original contributions presented in the study are included in the article/supplementary material, further inquiries can be directed to the corresponding authors.

## Ethics statement

The studies involving human participants were reviewed and approved by Research Ethics Committee of the Shanghai Pudong New Area Mental Health Center and Tongji University Mental Health Center (No. PDJWLL2019008). The patients/participants provided their written informed consent to participate in this study.

## Author contributions

YF, HF, TJ, and LZ recruited participants and collected the data. QW recommended the scales used in the study. YS, JT, and YY completed the scales data entry, analyzed the data of scales, and wrote the manuscript. YY and QW designed the study and revised the paper. All authors have read and approved the manuscript.

## Funding

This study was supported by grants from the following institutions: Shanghai Pudong New Area Science and Technology Development Fund (No. PKJ2021-Y78), Construction of characteristic disciplines of Public Mental Health of Shanghai Pudong New Area (No. PWYgts2021-01), and the Outstanding Clinical Discipline Project of Shanghai Pudong (No. PWYgy2021-02).

## Conflict of interest

The authors declare that the research was conducted in the absence of any commercial or financial relationships that could be construed as a potential conflict of interest.

## Publisher's note

All claims expressed in this article are solely those of the authors and do not necessarily represent those of their affiliated organizations, or those of the publisher, the editors and the reviewers. Any product that may be evaluated in this article, or claim that may be made by its manufacturer, is not guaranteed or endorsed by the publisher.
